# Neuroprotective and probiotic potential of *Lactiplantibacillus plantarum* AM2 in cognitive impairment

**DOI:** 10.1038/s41598-025-06103-9

**Published:** 2025-06-20

**Authors:** Walid A. Lotfy, Amira M. Ali, Heba M. Abdou, Khaled M. Ghanem

**Affiliations:** 1 Department of Microbiology, Faculty of Dentistry, Pharos University in Alexandria, Alexandria, Egypt; 2 Department of Botany and Microbiology, Faculty of Science, Alexandria University, Alexandria, Egypt; 3 Department of Zoology, Faculty of Science, Alexandria University, Alexandria, Egypt

**Keywords:** Acetylcholine, Alzheimer disease, Cognitive enhancement, *Lactiplantibacillus plantarum*, Probiotic, Biotechnology, Industrial microbiology

## Abstract

This study aimed to isolate and characterize an acetylcholine-producing probiotic strain and to evaluate its potential anti-Alzheimer properties in D-galactose rat model. Nine *Lactobacillus* isolates were isolated and purified on MRS agar, with six demonstrating acetylcholine (ACh) production capability. Among these, *Lactiplantibacillus* sp. AM2 showed the highest ACh production (78.4 pg/mL) and was identified as *Lactiplantibacillus plantarum* through morphological, biochemical, 16S rRNA sequencing and matrix-assisted laser desorption ionization time of flight mass spectrometry (MALDI-TOF MS). The *in-vitro* probiotic characterization of *Lactiplantibacillus plantarum* AM2 included tolerance testing for acidic conditions and bile salts, viability under simulated digestive conditions, antimicrobial activity assessment, and sensitivity testing to antibiotics. Behavioral and biochemical tests were conducted in a D-galactose-induced cognitive impairment rat model to evaluate cognitive performance, serum glucose levels, oxidative stress markers, and antioxidant capacity. Histopathological evaluations of the hippocampus were also performed. *Lactiplantibacillus plantarum* AM2 demonstrated strong tolerance to acidic conditions and bile salts, maintaining over 80% viability after exposure to simulated gastric and pancreatic juices. The strain showed hydrophobicity toward hexadecane, octane, and xylene. It also exhibited significant antimicrobial activity against pathogenic bacteria and was sensitive to multiple antibiotics. In cognitive impairment tests, rats administered with *Lactiplantibacillus plantarum* AM2 showed reduced time latency in the Morris Water Maze, suggesting cognitive enhancement. Biochemical analyses revealed improvements in serum glucose levels, reduced oxidative stress markers, and increased glutathione and total antioxidant capacity. Histopathological analyses showed mitigation of hippocampal damage, promoting recovery of normal cellular architecture. These findings highlight *Lactiplantibacillus plantarum* AM2 as a promising probiotic candidate with neuroprotective potential and notable ACh production. Further research is warranted to explore its application in therapeutic contexts.

## Introduction

Maintaining a healthy gut microbiota is crucial for supporting the immune system and cognitive emotional balance as it produces numerous biologically active metabolites, thus influencing the communication between the gut microbiota and the brain^1^. Germ-free mice studies have revealed that the presence of gut bacteria can impact the development of the immune, endocrine, and nervous systems^2^. The microbiome-gut-brain axis (MGBA) is a bidirectional communication system that connects gut bacteria with the brain through neurological, immunological, endocrine, and metabolic pathways^3^. While the gut and brain are physically distinct, several mechanisms have been identified that allow the gut microbiome to communicate with the central nervous system (CNS)^4^. These include the modulation of the immune system, vagus nerve, enteric nervous system (ENS), neuroendocrine system, and circulatory system, facilitated by the production of neuroactive compounds, metabolites, and hormones^5^. The MGBA can be seen as a multifunctional network, where different systems can participate in bidirectional communication^6^.

The gut microbiome can generate neuroactive compounds, including neurotransmitters and their precursors, which have the potential to influence the levels of related neurotransmitters and their precursors within the brain^7^. The gut microbiota produces various neurotransmitters, including both excitatory types like glutamate, acetylcholine (ACh), and dopamine, as well as inhibitory ones such as γ-aminobutyric acid, glycine, and serotonin^8^. Neurotransmitters play a crucial role in regulating diverse cognitive and behavioral processes, such as motor function, emotional regulation, learning, and memory formation^9^.

Alzheimer disease (AD) is a degenerative neurological condition that progresses over time, characterized by the presence of extracellular Aβ plaques and intracellular neurofibrillary tangles, which are composed of hyperphosphorylated tau and loss in cholinergic synapses^10^. AD is the most common form of dementia, affecting at least two-thirds of individuals aged 65 and older^11^. Cholinergic dysfunction is one of AD causes, this hypothesis is based on the early loss of cholinergic neurons in AD that leads to reduced level of ACh, underscoring the importance of ACh in cognitive processes^11^.

Numerous studies have mentioned the impact of gut microbiota dysbiosis in the pathophysiology of AD^12^. Several hypotheses have been suggested regarding the involvement of gut microbiota in various processes, such as direct microbial infection in AD^12^. Additionally, there are theories linking these microbes to immune system aging^13^. A clinical research study has also found that the gut microbiota of individuals with AD exhibits lower microbial diversity and alterations in bacterial levels, such as reduced levels of *Firmicutes* and *Bifidobacterium*, and elevated levels of *Bacteroidetes*^14^. It has been noted that as individuals age, the composition of gut microbiota changes, with an increase in *Proteobacteria* and a decrease in *Bifidobacteria*, as well as neuroprotective molecules such as short chain fatty acids (SCFAs)^15^. Furthermore, research has demonstrated a link between the decline in microbiome function, particularly genes responsible for producing SCFAs, and elevated levels of proinflammatory cytokines in older individuals without health issues^16^. There is a suggestion that the connection between age-related dysbiosis and neurological decline involves chronic low-grade inflammation, which serves as a common basis for various age-related pathologies, referred to as inflamm-aging^17^.

It has been demonstrated that in AD there is a significant decrease in the abundance of bacteria from the *Verrucomicrobia*, *Actinobacteria*, *Firmicutes*, and *Proteobacteria* genera^18^. It is also common to notice a rise in genera *Tenericutes* and *Bacteroidetes*^14^. This unique microbial composition promotes the accumulation of Aβ in the brain^19^.

Changes in gut microbiota can lead to the colonization of internal pathogens and increased permeability of the intestines which could potentially interfere with the gut-brain axis function. Certain opportunistic bacteria in the intestines, such as *Escherichia coli*, *Mycobacterium* spp., *Salmonella* spp., *Staphylococcus aureus*, *Klebsiella pneumoniae*, and *Streptococcus* spp. can release microbial exudates and lipopolysaccharide (LPS), which are also recognized as immunogenic components of amyloids^20^. LPS is present in Aβ within the amyloid plaque, indicating that bacterial components may travel from the gut to the brain through bloodstream, potentially worsening Aβ−42 deposition in AD^18^. As a result, specific parts of the brain like the cerebellum and hippocampus, may suffer from decreased functionality^18^.

The current study aimed to i) investigate the putative characteristics of *Lactiplantibacillus plantarum* AM2 as a new probiotic strain and ii) evaluate the effect of *Lactiplantibacillus plantarum* AM2 in the prevention of cognitive deficits of AD in D-galactose-induced rats.

## Materials and methods

### Isolation sources of *Lactiplantibacillus *spp.

Samples were purchased from the local market including, mixed vegetable pickle, cabbage, yogurt, and rayeb. Sauerkraut was prepared by adding 20 g of sodium chloride to alternate layers of 1 kg shredded cabbage. The layers were pressed down and left for 10 days at room temperature. The isolate AM2, which demonstrated the highest ACh production, was obtained from Sauerkraut.

### Indicator pathogenic microorganisms

The following pathogens were used as indicator strains to determine the antimicrobial activity: *Salmonella typhimurium* (ATCC 14028), *Staphylococcus aureus* (ATCC 29223), *Pseudomonas aeruginosa* (ATCC 27853), *Enterococcus faecalis* (ATCC 29212), *Escherichia coli* (ATCC 25922), and *Candida albicans* (ATCC 10231).

### Media and chemicals

The culture media used throughout the current study were prepared according to the manufacturer’s instructions. The following media (g/L) were used. *De man, Rogosa and Sharpe medium (MRS) broth* (Merck KGaA, Darmstadt, Germany): Peptone from casein, 10.0; meat extract, 8.0; yeast extract, 4.0; D (+)-glucose, 20.0; dipotassium hydrogen phosphate, 2.0; tween 80, 1.0; di-ammonium hydrogen citrate, 2.0; sodium acetate, 5.0; magnesium sulfate, 0.2; and manganese sulfate, 0.04. The medium was supplemented with 25 mg choline (HiMedia, India)/L of medium as a precursor for ACh production^21^. *Nutrient broth (NB) medium* (HiMedia, India): Peptone, 10.0; beef extract, 10.0; and sodium chloride, 5.0. *Nutrient agar (NA) medium* (HiMedia, India): Peptone, 10.0; beef extract, 10.0; sodium chloride, 5.0; and agar, 15. *Müller-Hinton agar (MHA) medium* (HiMedia, India): Beef extract, 2; casein hydrolysate, 17.5; starch, 1.5; and agar, 17. All chemicals used in the present work were of analytical grade. Simulated gastric juice (3.0 mg pepsin/mL) was prepared by adding 0.09 g pepsin to 30 mL sodium chloride solution (0.5%, w/v). Pancreatic juice (1.0 mg pancreatin/mL) was prepared by adding 0.02 g pancreatin to 20 mL sodium chloride solution (0.5%, w/v). The following kits were used in the biochemical analysis: Glucose assay Kit (Biodiagnostic, Egypt), ELISA kit (Cell Biolabs, Inc, USA) for advanced glycation end products (AGEs) assay, Glutathione (GSH) assay kit (Biodiagnostic, Egypt), Malondialdehyde (MDA) assay kit (Biodiagnostic, Egypt), total antioxidant capacity (TAC) assay Kit (Biodiagnostic, Egypt), ELISA kit (Cloud- Clone Crop, USA) for acetylcholine (ACh) assay, ELISA kit (BT-laboratory, China) for acetylcholinesterase assay (AChE), ELISA kit (BT-laboratory, China) for nuclear factor-kappa B (NF-κB) assay, ELISA kit (BT-laboratory, China) for tumor necrosis factor-alpha (TNF-α) assay, ELISA kit (BT-laboratory, China) for interleukin-6 (IL-6) assay, ELISA kit (Cloud- Clone Crop, USA) for interleukin 1-beta (IL-β) assay, and ELISA kit (Novus Biologicals, USA) for amyloid beta1-42 (Aβ1–42) assay.

### Isolation and purification of *Lactiplantibacillus *spp.

An aliquot of 1 mL from rayeb, yogurt, pickle, or sauerkraut was transferred to a tube containing 9 mL sterile saline (0.9%) and serially diluted from 10^–1^ to 10^–6^. A portion of 0.1 mL from each dilution was plated onto MRS agar plates and was then spread using a sterile spreader. The plates were incubated anaerobically in an anaerobic jar for 24–48 h at 37°C. After incubation, the colonies were randomly isolated and purified on MRS agar plates using streaking technique.

### Seed inoculum

A loopful of *Lactiplantibacillus* sp. pure culture was inoculated onto the surface of MRS agar and was then incubated at 37 °C under anaerobic conditions in an anaerobic jar for 24 h. A seed culture of 10^9^ (9 log) CFU/mL was prepared by adjusting the optical density (OD_600_) to 1.28 using spectrophotometer (T80 + UV/VIS PG instrument LTD, UK), which corresponds to the mid-logarithmic phase of growth. A standardized inoculum (1.0%) was used throughout the experiments. The CFU/mL was confirmed using a standard curve correlating OD_600_ with viable counts.

### Screening and selection of a promising *Lactiplantibacillus *sp. capable of producing acetylcholine

MRS broth was transferred to an Erlenmeyer flask upto a final volume of 50 mL. An aliquot of 0.5 mL of seed inoculum, previously standardized to 10⁹ CFU/mL in mid-log phase, was inoculated to the culture then incubated anaerobically in an anaerobic jar at 37 °C for 24 h and the concentration of ACh was measured after incubation.

### Determination of acetylcholine

ACh was measured quantitatively *in* *vitro* using competitive inhibition enzyme immunoassay kit. Briefly, in a microplate a competitive inhibition reaction was set between unlabeled ACh in standards or control or cell free supernatant (50 μL) and biotin labeled ACh (50 μL) with the precoated antibody specific to ACh. After incubation at 37 °C for 1 h, the unbound conjugate was washed off 3 times using 350 μL washing solution. Next, an aliquot of 100 μL of avidin conjugated to horseradish peroxidase (HRP) was added to each microplate well and incubated at 37 °C for 30 min. The washing process was repeated for 5 times using 350 μL washing solution. Next, an aliquot of 90 μL of substrate solution was added to each microplate well and incubated at 37 °C for 20 min. After incubation, 50 μL of stop solution was added to each microplate well then, the intensity of the developed color which is reverse proportional to the concentration of ACh in the samples was measured using a plate reader (Microlisa—Micro Lab Instruments, India) at 450 nm. A standard curve ranged from 12.35 to 100 pg/mL was conducted with the log of ACh concentration on the y-axis and absorbance on the x-axis. The average of triplicate readings for each standard, control, and samples was calculated. In the case of dilution of samples, the concentration read from the standard curve was multiplied by the dilution factor.

### Identification of bacterial isolates

The obtained isolates were stained using Gram stain, this was followed by microscopic examination (Labovision, AXE 2000, India). The isolate that showed the highest production of ACh underwent molecular sequencing (ABI PRISM 3730xl DNA sequencer), biochemical identification (Biologsystem VITEK 2 Version 7.01), and matrix-assisted laser desorption ionization time of flight mass spectrometry (MALDI-TOF MS) proteomic identification (SAI, LT2 +, UK).

#### Molecular identification using 16S rRNA sequence

Sequencing of the 16S rRNA was performed according to a previous study^22^. Deoxyribonucleic acid (DNA) was isolated from the selected isolate, then the 16S rRNA was amplified by polymerase chain reaction (PCR) using universal primers. The forward primer was 5’-AGA GTT TGA TCC TGG CTC AG-3’ and the reverse primer was 5’ -ACG GCT ACC TTG TTA CGA CTT-3’. The PCR mixture consists of 25 μL My Taq Red Mix, 8 μL DNA template, 1 μL (20 Pico mol) for each primer, and 15 μL nuclease free water. The PCR was carried out for 35 cycles in 94 °C for 45 s, 56 °C for 45 s and 72 °C for 1 min. After completion, a fraction of the PCR mixture was examined using agarose gel electrophoresis, and the remnant was purified using Gene JET PCR purification Kit (Thermo K0701). DNA sequences were obtained using an ABI PRISM 3730XL DNA sequencer. The PCR product was sequenced using the same PCR primers. Blast program was used to assess the DNA similarities and multiple sequence alignment was performed using BioEdit software version 7.5.0.3^23^. The phylogenetic tree was displayed using Mega 6.06^24^.

#### MALDI-TOF MS proteomic based identification

Proteomic spectra generated by MALDI-TOF MS were compared against the reference spectra using MALDI-TOF Bactoscreen software version 2.4 to obtain identification with a confidence score^25^.

### Evaluation of the probiotic characteristics of *Lactiplantibacillus *sp.

#### Acid tolerance

Acid tolerance experiment was carried out according to a previously reported method^26^. A seed culture of 10^9^ CFU/mL of *Lactiplantibacillus* sp. was prepared and an inoculum size of 1.0% was transferred to MRS broth adjusted to pH 2.0 and pH 3.0 using 3.0 M hydrochloric acid. Another Erlenmeyer flask containing MRS medium adjusted to pH (5.7 ± 0.1) served as a negative control. The cultures were mixed well then incubated anaerobically in an anaerobic jar at 37°C. The count of *Lactiplantibacillus* sp. on MRS agar medium was monitored at time intervals of 0, 60, 120 and 180 min using pour plate method^27^. The plates were incubated anaerobically in an anaerobic jar at 37 °C for 48 h then the bacterial colonies were counted and converted into CFU/mL. The survival rate (%) was calculated according to the following equation^1^:

$$ {\text{Survival }}\left( {\text{\%}} \right) = \frac{{\log {\text{CFU}}/{\text{mL after treatment}}}}{{{\text{Log CFU}}/{\text{mL before treatment}}}} \times 100 $$

#### Bile tolerance

The bile tolerance experiment was performed according to a previously reported study^28^ with some modifications. A seed culture of 10^9^ CFU/mL of *Lactiplantibacillus* sp. was prepared and an inoculum size of 1.0% was transferred to MRS broth containing various ox-bile (10%) concentrations viz. 0.3, 0.5 and 1.0% (v/v). The cultures were incubated anaerobically in an anaerobic jar at 37 °C for 4 h then the count (CFU/mL) of *Lactiplantibacillus* sp. on MRS agar medium was determined after incubation at 37 °C for 48 h. The survival rate (%) was calculated as mentioned previously.

#### Simulated gastrointestinal tract tolerance

The *in-vitro* determination of *Lactiplantibacillus* sp. viability was carried out according to a previous study^29^ under conditions similar to gastrointestinal tract. Overnight culture of *Lactiplantibacillus* sp. was centrifuged at 5000 rpm for 15 min then washed twice with phosphate buffer saline (PBS) pH 7.0. A bacterial suspension (10^9^ CFU/mL) was prepared by suspending *Lactiplantibacillus* sp. in PBS. Simulated gastric juice (3.0 mg pepsin/mL) and simulated pancreatic juice (1.0 mg pancreatin/mL) were adjusted to pH 3.0 and 8.0, respectively. A mixture of 1.0 mL simulated gastric juice or simulated pancreatic juice, 0.3 mL sodium chloride solution (0.5%, w/v) and 0.01 mL of bacterial suspension was added to a sterile test tube. A negative control was conducted by using 1.0 mL sodium chloride solution (0.5%, w/v) instead of the simulated gastric or pancreatic juice. The tubes were mixed well using a vortex mixer (Assistent Reamix 2789) then incubated for 0, 180 and 240 min at 37°C. The survival rate (%) was calculated as mentioned previously.

#### Adhesion to stainless steel chips

Adhesion to stainless steel experiment was employed to study the potential adhesion of *Lactiplantibacillus* sp. on smooth stainless steel surfaces which reduce the bacterial adhesion properties^30^. The study was performed according to a method previously described in a reported study^31^ with some modifications. Briefly, the stainless steel chip (2.2 × 2.2 cm) was cleaned and sterilized to make them grease as well as microbes free. The individual chip was inoculated to 40 mL MRS broth containing *Lactiplantibacillus* sp. (10^9^ CFU/mL) and incubated anaerobically in an anaerobic jar over night at 37°C. After the incubation time, the chips were removed carefully and fixed in 4 formaldehyde (F): 1 gluteraldehyde (G) fixative for 24 h. After fixation, the chips were removed from the fixative (4F:1G) and washed by distilled water. Thenceforward, the chips were dehydrated in graded series of alcohol from 30 to 70%, dried in air, and coated with gold to be examined under JSM-IT200 series scanning electron microscope (SEM).

#### Cell surface hydrophobicity

The cell surface hydrophobicity of *Lactiplantibacillus* sp. was conducted according to a previously reported method^32^. Briefly, 2 tubes each containing a mixture of 5.0 mL bacterial suspension (10^9^ CFU/mL) and 1.0 mL hydrocarbon (xylene or hexadecane or octane) were vortexed using a vortex mixer (Assistent Reamix 2789) for 2 min. The lower aqueous phase was removed carefully by sterile Pasteur pipette from one tube, then the initial absorbance was measured at 600 nm by using a spectrophotometer (T80 + UV/VIS PG instrument LTD, UK). The other tube was incubated for 1 h at 37°C. After incubation, the lower aqueous phase was similarly removed, and the final absorbance was measured at 600 nm. The percentage of hydrophobicity (H%) was calculated according to the following equation:

$$H\% = \frac{{A_{^I}{-}A_{^F } }}{A_{^I}} \times 100$$

where the $A_{^I}$ and $A_{^F}$ are the initial absorbance and the final absorbance at 600 nm, respectively.

#### Antagonistic activity against pathogens

The antimicrobial activity of *Lactiplantibacillus* sp. was investigated against the following pathogenic indicators: *Salmonella typhimurium* (ATCC 14028), *Staphylococcus aureus* (ATCC 29223), *Pseudomonas aeruginosa* (ATCC 27853), *Enterococcus faecalis* (ATCC 29212), *Escherichia coli* (ATCC 25922), and *Candida albicans* (ATCC 10231). The pathogenic indicators were cultured overnight on NA media and then suspended in saline solution to prepare 10^8^ CFU/mL suspension. A sterile cotton swab was immersed in each suspension and used to inoculate the surface of MHA plate in a zigzag style along each of its four sides, as well as around the edges. Wells were cut using a sterile 4 mm cork borer and 50 μL of *Lactiplantibacillus* sp. liquid culture filtrate were transferred into each well after filtration using 0.22 µm sterile nitrocellulose syringe filters^33^. The plates were incubated at 37 °C for 48 h. After the incubation period, the absolute unit (AU) was calculated using the following formula:

$$AU = \frac{x2}{{y2}}$$

where (*x*) is the clear zone radius around each well and (*y*) is the well radius in mm.

To rule out acid inhibition, a portion of the supernatant samples obtained from *Lactiplantibacillus* sp. was neutralized to pH 6.5 by NaOH (1M) for the measurement of antimicrobial activity.

#### Susceptibility to antibiotics

The antibiotic susceptibility profile of the *Lactiplantibacillus* sp. was determined according to the guideline of Clinical and Laboratory Standard Institute^34^. Briefly, various dilutions of antibiotics ranged from 512 to 0.125 μg/mL were added to MRS broth. This was followed by inoculation of *Lactiplantibacillus* sp. (1%, v/v) to MRS broth and incubation at 37 °C for 24 h. The following antibiotics were used to test the antibiotic resistance pattern of *Lactiplantibacillus* sp.: amoxicillin, amoxicillin/clavulanic acid, piperacillin, piperacillin/tazobactam, ceftazidime, cefepime, aztreonam, imipenem, meropenem, amikacin, gentamicin, tobramycin, ciprofloxacin, minocycline, and trimethoprim/sulfamethoxazole (Oxoid, USA). The minimum inhibitory concentration (MIC) of each antibiotic for the strain was measured by VITEK 2 Version 7.01.

### Evaluation of the cognitive function in Alzheimer’s disease induced Wistar albino rats

#### Probiotic dosage preparation for animal experiment

The probiotic dosage was prepared fresh daily by centrifugation of bacterial cells at 8000 rpm for 5 min^35^, followed by washing the pellets twice with sterile saline solution, then adjusting the concentration to 10^9^ CFU/mL.

#### Animal and experimental design

The experimental protocol of the animal study was approved by the Alexandria University Egypt Institutional Animal Care and Use Committee (ALEXU-IACUC approval number: AU04230530302). All experiments were performed in accordance with relevant guidelines and regulations. All methods are reported in accordance with ARRIVE guidelines. Twenty-four adults male Wistar albino rats weighting 180 ± 20 g were obtained from animal house at Pharos University, Alexandria, Egypt. Animals were housed in stainless steel wire cages placed in a ventilated animal room maintained at 21 ± 3 °C with 12 h dark and light cycle and kept on basal diet and tap water ad libtium. After one week of acclimation, animals were randomly divided into four equal groups, each of 6 rats as follow: Group 1 (Control): rats of this group were orally received 2 mL/190 g body weight saline (0.9% w/v) by gavage, daily for 8 weeks. Group 2 (*Lactiplantibacillus* sp.—treated group): rats of this group were orally received 2 mL/190 g body weight with concentration of 10^9^ CFU/mL daily for 8 weeks^36^. Group 3 (D-galactose—treated group): Rats of this group were orally received 0.5 mL of D-galactose suspended in saline at a dose of 200 mg/kg body weight for 8 weeks. Group 4 (D-galactose + *Lactiplantibacillus* sp.—treated group): Rats of this group were orally administrated 0.5 mL/190 g of D-galactose after 30 min of *Lactiplantibacillus* sp. administration daily for 8 weeks.

#### Behavioral test

The behavioral test was performed in the 7^th^ week of the experiment, between 9 am and 2 pm. The Moris water maze (MWM) was performed to evaluate the spatial memory that depends on the hippocampus^37^. The experiment applied in grey circular pool with diameter 1.5 m and depth 55 cm and filled with clear water 19 ± 2°C. The water pool was divided into 4 equal quadrants northwest (NW), northeast (NE), southeast (SE), and southwest (SW). The circular platform with 10 cm diameter and 22 cm depth was placed in center of SW quadrant, during the acquisition phase the platform appeared 2 cm above the clear water. The rats were trained 3 times once in each quadrant (NW, NE, SE, SW), after the rats reach the platform they were allowed 15 s before the next trial. Then the water was opaqued with white powder (starch). The platform emerged 2 cm under water and rats were trained 3 times, once in each quadrant. In the probe phase the platform was removed, and rats start their test from north (N) position, the latency time was recorded.

#### Blood and brain collection

At the end of the experimental period, the rats underwent a 12-h fasting period before blood and brain tissue sampling. Subsequently, they were subjected to euthanasia using intraperitoneal mixture of ketamine (100 mg/kg) and xylazine (5 mg/g). While under the influence of anesthetic and sacrificed through cervical dislocation. Blood samples were obtained in both regular test tubes and test tubes coated with the anticoagulant ethylene diamine tetra acetic acid (EDTA) for the purpose of assessing glucose and advanced glycation end products (AGEs). The tubes were promptly placed on ice. Serum samples were produced and stored at −20 °C in a pyrogen-free epindorff for biochemical analysis. Animals’ brains were dissected to isolate the hippocampus; the left hemispheres were extracted to evaluate biochemical markers, while the right hemispheres were fixed in 10% formalin and processed for microscopical studies.

#### Tissue preparation

For biochemical investigation, the left hippocampus from each rat, (amounting to one-fourth of a gram), was individually homogenized in 2 mL of a cold buffer (50 mM potassium phosphate adjusted to pH 7.5 and 1 mM EDTA) per gram of tissue. This homogenization process was carried out using a tissue homogenizer (Tekmar model TR-10, West Germany). The resulting homogenate was then subjected to centrifugation at 4000 rpm for 15 min, employing a cooling centrifuge (Hettich model EBA 12R, Germany). Subsequently, the supernatants were collected and stored at −80 °C for the determination of the biochemical parameters.

### Biochemical parameters analysis

#### Assessment of serum glucose level

According to a previously reported method^38^, glucose can be oxidized by glucose oxidase (GOD) to gluconic acid with the formation of H_2_O_2_. In the presence of peroxidase enzyme (POD), phenol and 4-aminoantipyrine (AAP) can be oxidized by H_2_O_2_ forming a red quinoneimine dye which is comparative to the level of glucose in the sample. One mL of the working reagent was added to 0.01 mL of the serum sample or standard. The mixture was thoroughly blended and incubated at 37 °C for 10 min. The absorbance (A) values of the sample and standard were measured at 510 nm against reagent blank which contained 0.01 mL distilled water instead of the sample.

$$\tt {\text{Serum glucose level }}\left( {{\text{mg}}/{\text{dL}}} \right){ } = \frac{{A{\text{ sample}}}}{{A{\text{ standard}}}} \times 10$$

#### Determination of serum advanced glycated end products (AGEs) concentration

Rat AGEs ELISA Kit (A80246) employs the sandwich enzyme immunoassay technique was used according to the manufacturer’s protocol for the quantitative measurement of rat AGEs in serum, plasma, and tissue homogenates. A capture antibody binds AGEs, followed by a detection antibody and horseradish peroxidase (HRP)-streptavidin conjugate. The colorimetric signal generated is proportional to the AGEs concentration. The concentration of AGEs was calculated by reading the absorbance at 450 nm in a microplate reader and referring to the standard curve created by plotting the average absorbance for each standard on the y-axis against the standard concentrations on the x-axis.

#### Determination of lipid peroxidation end product (Malondialdhyde; MDA) concentration in hippocampus tissues

Lipid peroxidation was measured as thiobarbituric acid (TBA) that reacts with MDA in acidic medium at temperature of 95 °C for 30 min to form thiobarbituric acid reactive product. The absorbance of resultant pink product was measured at 534 nm^39^. The absorbance (A) of sample was measured against blank, and a standard solution was measured against distilled water at 534 nm.

$${\text{MDA}}\,{\text{in}}\,{\text{tissue}}\,{\text{sample}}\,\left( {{\text{n}}\,{\text{mol}}/{\text{mg}}\,{\text{protein}}} \right) = \,\frac{{{\text{A}}\,{\text{sample}}}}{{{\text{A}}\,{\text{standad}}}} \times 10/{\text{g}}\,{\text{protein}}\,{\text{used}}$$

Where, A _sample_ is the absorbance of sample, and A _standard_ is the absorbance of standard.

#### Determination of reduced glutathione (GSH) level in hippocampus tissue

GSH can react with Ellman’s reagent; DTNB (5,5’-dithio-bis-2-nitrobenzoic acid) forming a yellow colored 2-nitro-5-thiobenzoic acid product^40^. The proteins were precipitated using 0.1 mL of 4% sulfosalicylic acid in 0.1 mL of each tissue extract. The samples were kept at 4 °C for 1 h then centrifuged at 3000 rpm for 10 min at 4°C. One mL from each supernatant was added to 2.7 mL phosphate buffer (0.1 M, pH 7.4) and 0.2 mL DTNB. The tubes of the standard and the blank experiments were prepared using 0.1 mL of standard GSH and distilled water respectively instead of the samples. The absorbance (A) of the samples and the standard were read against blank at 412 nm. GSH level was calculated according to the following equation:

$${\text{GSH level }}\left( {\upmu {\text{ mol}}/{\text{mg protein}}} \right) = \left[ {\frac{{\text{A sample}}}{{\text{A standard}}} \times {\text{Standard concentration}}} \right]{\text{/Total protein}}$$

where, A _sample_ is the absorbance of sample, and A _standard_ is the absorbance of standard.

#### Determination of total antioxidant capacity (TAC) in hippocampus tissues

TAC in the hippocampus was measured according to a previous study^41^. TAC in the hippocampus was determined by assessing the antioxidant ability of the sample to remove a significant portion of the provided exogenous H_2_O_2_. The remaining H_2_O_2_ was then quantified through an enzymatic reaction that converted 3,5-dichloro-2-hydroxylbenzenesulfonate into a colored product. The absorbance (A) of the blank and sample were measured against distilled water at 505 nm in 1 mL plastic cuvettes, and the total antioxidant capacity was calculated according to the following equation:

Total antioxidant concentration (mM/mg protein) = (A _Blank_ -A _Sample_) × 3.33 × dilution factor

Where: A_Blank_ is the absorbance of blank, and A _Sample_ is the absorbance of the sample.

#### Determination of hippocampus acetylcholine level

The assay utilizes a competitive inhibition enzyme immunoassay method. A monoclonal antibody specific to ACh has been pre-coated onto the microplate surface. A competitive inhibition reaction was initiated between biotin-labeled ACh and unlabeled ACh for binding to the pre-coated ACh-specific antibody. A logarithmic standard curve was generated, with the logarithm of ACh concentration (pg/mg protein) plotted on the x-axis and absorbance (at 450 nm) on the y-axis.

#### Determination of acetylcholine esterase (AChE; EC 3.1.1.7) activity in hippocampus tissue

This ELISA kit employs the Sandwich-ELISA technique. The micro-ELISA plate is pre-coated with an antibody specific to rat AChE. Standards or samples were added to the plate wells and combined with the specific antibody. Next, a biotinylated detection antibody specific for rat AChE and an Avidin-HRP conjugate were added sequentially to each well and incubated, then the OD was measured at 450 nm. Duplicate readings for each standard and sample were averaged, and the average zero standard optical density was subtracted. Finally, a standard curve was plotted with standard concentration on the x-axis and OD values on the y-axis.

#### Determination of NF-κB in hippocampus of rats

This ELISA assay is based on the Sandwich-ELISA method. The micro-ELISA plate has been pre-coated with an antibody specific to rat NF-κB. Samples were added to the micro-ELISA plate wells and incubated with the specific antibody. Then, a biotinylated detection antibody specific for rat NF-κB-p65 and an Avidin-HRP conjugate were sequentially added to each micro-plate well. The OD which is proportional to the concentration of rat NF-κB was measured spectrophotometrically at 450 nm. The duplicate readings for each standard and sample were averaged, and the average zero standard OD was subtracted. A standard curve was plotted with standard concentration on the x-axis and OD values on the y-axis.

#### Determination of tumor necrosis factor alpha (TNF-α) in hippocampus tissue

TNF-α levels were quantified using an ELISA assay^42^. for the purpose of measuring rat TNF-α in tissue supernatants. The mean absorbance values were calculated for each set of duplicate standards, controls, and samples, with the average blank (zero standard) OD subtracted. A standard curve was then generated, with standard concentration plotted on the x-axis and OD at 450 nm on the y-axis. The TNF-α concentration in each sample was subsequently determined by interpolation from this standard curve.

#### Determination of interleukin-6 (IL-6) in hippocampus tissue

Rat IL-6 levels were determined using a sandwich ELISA kit, following a method from a previous study^43^ and the OD was measured at 450 nm. The averaged duplicate readings for each calibrator and sample were adjusted by subtracting the average OD of the zero calibrator. A logarithmic calibration curve was then constructed, with IL-6 concentration plotted on the x-axis and the corresponding OD values on the y-axis.

#### Determination of interleukin-1β (IL-1β) in hippocampus tissue

A rat IL-1β ELISA kit was utilized to quantitatively measure the concentration of IL-1β in the tissue supernatant. The analysis was conducted following a method described in a previous study^43^ and the OD of the plate was read at 450 nm. A standard curve was generated by plotting the adjusted mean OD for each reference standard against its corresponding concentration in pg/g tissue on a logarithmic scale. The adjusted mean OD was calculated by subtracting the average OD of the negative control from the average of each observed OD.

#### Determination of hippocampus amyloid beta peptide 1–42 (Aβ1–42)

The rat Aβ1–42 concentrations in the supernatant were quantitatively determined using a method previously described in a reported study^44^. This assay employs a quantitative sandwich enzyme immunoassay technique. Antibody specific for Aβ1–42 was pre-coated onto a microplate. Standards and samples were pipetted into the wells, and any Aβ1–42 present was bound by the immobilized antibody. After removing any unbound substances, a biotin-conjugated antibody specific for Aβ1–42 was added to the wells. Following a washing step, avidin conjugated HRP was added to the wells. A subsequent wash to remove any unbound avidin-enzyme reagent was followed by the addition of a substrate solution, which developed color in proportion to the amount of Aβ1–42 bound in the initial step. The color development was stopped, and the intensity of the color was measured at 450 nm. A calibration curve using linear regression curve-fit was constructed, and the absorbance values of the diluted samples within the standard curve range were used to calculate the concentration of rat Aβ1–42 in the tested sample, which was then multiplied by the sample dilution factor.

#### Determination of hippocampal caspase-3 (CASP-3) concentration

The microplate in this assay kit was pre-coated with an antibody specific to CASP-3. Standards or samples were added to the appropriate microplate wells along with a biotin-conjugated antibody specific to CASP-3. Subsequently, avidin conjugated to HRP was introduced to each microplate well. Following the addition of 3,3′,5,5′-Tetramethylbenzidine (TMB) substrate solution, only the wells containing CASP-3, biotin-conjugated antibody, and enzyme-conjugated avidin exhibited a color change. The OD was measured at 450 nm for each sample and was subtracted from the blank well. The CASP-3 concentration in each sample was then interpolated from the standard curve.

### Histopathological study

Small pieces of hippocampus tissue were initially preserved in a 10% formalin solution. The standard histological procedure, involving paraffin-wax sectioning and staining with hematoxylin and eosin (H and E) as described in a previous study^45^, was employed for this analysis. The fixed tissue specimens underwent a dehydration process, wherein they were sequentially immersed in ascending concentrations of ethyl alcohol until they reached absolute alcohol over the course of one hour. Subsequently, they underwent a three-stage transition in xylol, with each stage lasting five minutes, and were then immersed in a mixture of paraffin wax and xylol (1:1 ratio) for a duration of 10 min. Following this, they were placed in wax at a temperature of 65 °C, with three transitions, each lasting approximately two hours. Sections measuring 5 μm in thickness were precisely sectioned using a microtome and affixed onto glass slides. One set of slides were meticulously prepared for further examination. This set was subjected to staining with H and E, facilitating subsequent histological analysis.

### Statistical analysis

Statistical analysis of the behavioral test and biochemical parameters analysis was performed using SPSS-16 software by applying one-way analysis of variance (ANOVA) test. The significance threshold was set at p < 0.05 for the 4 groups (n = 24).

## Results

### Screening for acetylcholine producer *Lactiplantibacillus *spp.

As described previously under the materials and methods section, *Lactiplantibacillus* strains were isolated from mixed vegetable pickle, sauerkraut, yogurt, and rayeb. Isolation was carried out on MRS agar plates and nine different bacterial isolates were isolated and purified following a previous study^46^. Each *Lactiplantibacillus* sp. was examined for the potential of its cell free supernatant to produce ACh. Only 6 *Lactoplantibacillus* spp. were capable of producing ACh. *Lactiplantibacillus* sp. AM2 showed the highest ACh production (78.4 pg/mL), therefore, it was selected as the most potent *Lactoplantibacillus* isolate. The strain exhibited stability with monthly subculturing on MRS agar plates, consistently producing ACh at a level of 78.1 pg/mL over 18 months.

### Identification of *Lactiplantibacillus *sp. AM2

#### Morphological characterization of *Lactiplantibacillus* sp. AM2

**Fig. 1:**
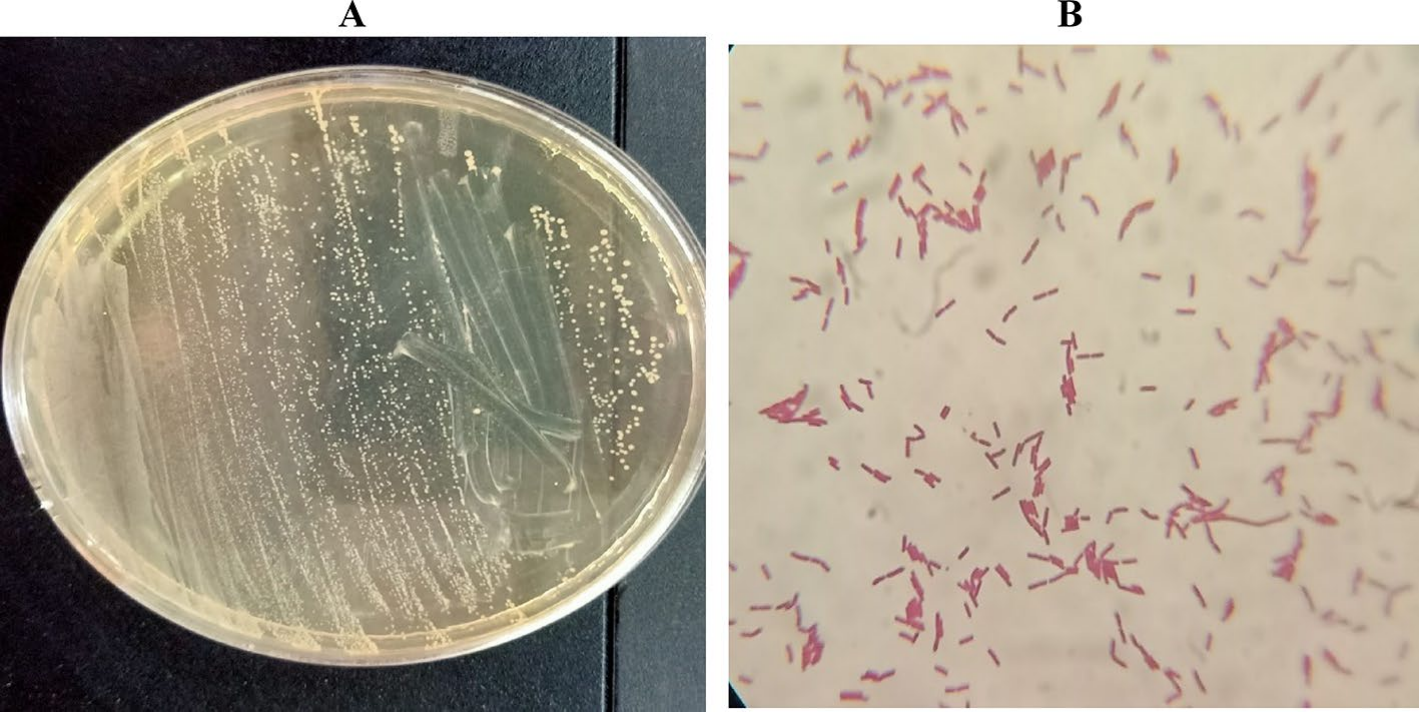
Cultural (**A**) and microscopic examination (**B**) of isolate AM2.

The growth of isolate AM2 on MRS agar showed slightly opalescent, medium amber in color colonies (Fig. 1A). The microscopic examination of isolate AM2 (Fig. 1B) revealed a Gram-positive, non-spore forming bacilli.

#### Biochemical characterization of isolate AM2

The biochemical profile of the *Lactiplantibacillus* isolate AM2 is presented in Table 1

**Table 1:** Biochemical profile of the *Lactiplantibacillus* isolate AM2.

Test	Result
D-amygdalin	-
Phosphatidylinositol phospholipase C	-
D-xylose	+
Arginine dihydrolase 1	+
Beta-galactosidase	-
Alpha-glucosidase	-
Ala-Phe-Pro-Arylamidase	-
Cyclodextrine	-
L-aspartate arylamidase	-
Beta galactopyranosidase resorufine	-
Alpha-mannosidase	-
Phosphatase	-
Leucine arylamidase	+
L-proline arylamidase	-
Beta-glucoronidase	-
Alpha-galactosidase	-
L-pyrrolydnyl-arylamidase	-
Beta-glucoronidase	-
Alanine arylamidase	+
Tyrosine arylamidase	-
D-sorbitol	-
Urease	-
Polymixin-B resistance	+
D-galactose	-
D-ribose	+
L-lactate alkalinisation	-
Lactose	-
N-acetyl-D-glucosamine	+
D-maltose	+
Bacitacin resistance	+
Novobiocin resistance	+
Growth in 6.5% NaCl	+
D-mannitol	+
D-mannose	-
Methyl-B-D-glucopyranoside	-
Pullulane	-
D-raffinose	-
Salicin	-
Sucrose	-
D-trehalose	-
Argininine dihydrolase 2	+
Optochin resistance	+

#### Molecular identification

**Fig. 2:**
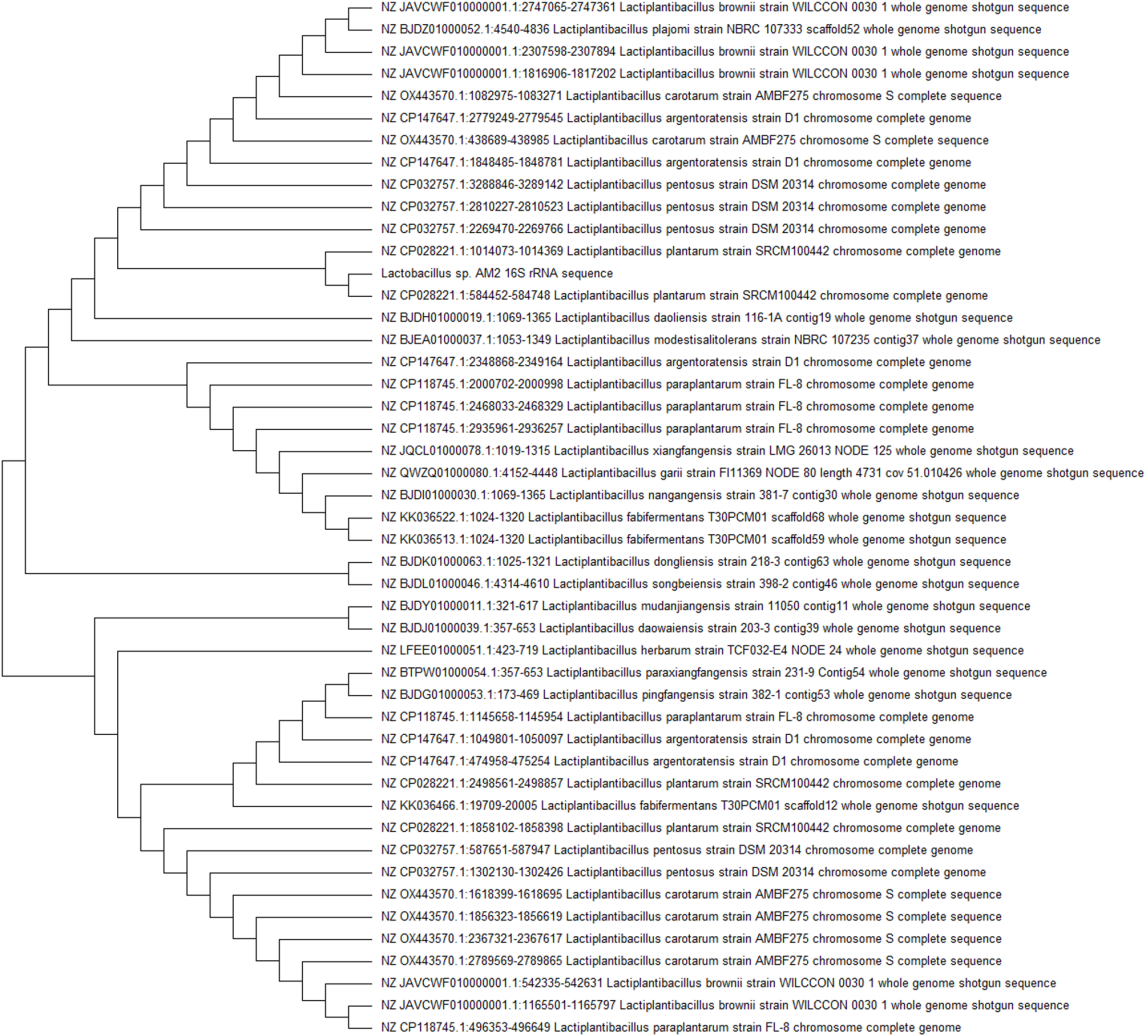
Phylogenetic relationships among the *Lactiplantibacillus* sp. AM2 and the most closely related bacterial species. The dendogram was generated by maximum likelihood method using Mega 6.06 software.

The amplified homologous 16S rRNA regions in the chromosomal DNA of *Lactiplantibacillus* isolate AM2 demonstrates that its rRNA fragment showed 100% identity to the homologous fragments of *Lactiplantibacillus plantarum*. The sequence has since been deposited in the GenBank under accession number ON142385. The phylogenetic position of the *Lactiplantibacillus* sp. AM2 among closely related bacterial species is shown as phylogenetic tree in (Fig. 2).

#### Matrix-assisted laser desorption ionization time of flight mass spectrometry (MALDI-TOF MS) identification

MALDI-TOF MS was used to compare the proteomic profiles of *Lactiplantibacillus* sp. AM2 cells to reference spectra. The AM2 strain showed high confidence genus and species identification based on the obtained score value (0.85) that matches *Lactiplantibacillus plantarum*. Hence, the isolate AM2 was identified as *Lactiplantibacillus plantarum* and has been deposited in the Egyptian Microbial Culture Collection Network, Alexandria, Egypt (accession number EMCCN 4080).

### Probiotic characterization of *Lactiplantibacillus plantarum* AM2

The resistance level of *Lactiplantibacillus plantarum* AM2 to the major components of gastric juice was evaluated *in-vitro* including tolerance to acidity, bile, pepsin and pancreatin.

#### Tolerance to acidity

*Lactiplantibacillus plantarum* AM2 was tested in a solution simulated to gastric juice at pH 2 and pH 3. As shown in Table 2, *Lactiplantibacillus plantarum* AM2 was able to maintain 37.8% and 81.7% of its viability after 3 h of exposure at pH 2 and pH 3, respectively. At pH 2, the strain retained 86.7% and 65.6% of viability after 1 h and 2 h of exposure, respectively. On the other hand, *Lactiplantibacillus plantarum* AM2 exhibited a higher resistance to pH 3 as the survival (%) was 94.9 and 88.4 after 1 h and 2 h of exposure.

**Table 2:** Survival of *Lactiplantibacillus plantarum* AM2 in gastrointestinal tract solution at different time intervals.

pH	Survival (%)			
	Control	1 h	2 h	3 h
2	100	86.7	65.6	37.8
3	100	94.9	88.4	81.7

#### Bile tolerance

*Lactiplantibacillus plantarum* AM2 survived well and showed high level of resistance to ox-bile at 0.3, 0.5 or 1.0% ox-bile in MRS broth (Table 3). The strain lost 3.93% and 8.68% of its viability after exposure to 0.5% and 1.0% ox-bile, respectively. The stain showed a trend of decreasing viability with increased ox-bile concentration.

**Table 3:** Survival of *Lactiplantibacillus plantarum* AM2 in the presence of different concentrations of ox-bile.

Survival (%)			
Control	0.3% ox-bile	0.5% ox-bile	1.0% ox-bile
100	100	96.07	91.32

#### Survival of *Lactiplantibacillus plantarum* AM2 under conditions simulating gastric and pancreatic juice

As shown in Table 4, *Lactiplantibacillus plantarum* AM2 was able to maintain 81.25% and 99.1% of its viability in the simulated gastric juice (pH 3) and pancreatic juice (pH 8), respectively, after incubation for 3 h. While after incubation for 4 h the AM2 strain was able to maintain 80.43% and 97.2% of its viability in the simulated gastric juice and pancreatic juice, respectively.

**Table 4:** Survival of *Lactiplantibacillus plantarum* AM2 in simulated gastric and pancreatic juice at different time intervals.

Survival (%)					
Simulated gastric juice			Simulated pancreatic juice		
Control	3 h	4 h	Control	3 h	4 h
100	81.25	80.43	100	99.10	97.20

#### Adhesion of *Lactiplantibacillus plantarum* AM2 to stainless steel chips

**Fig. 3:**
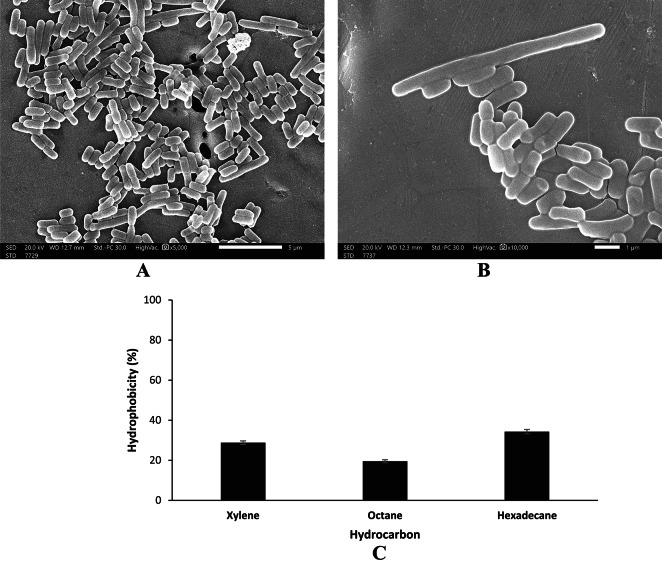
Cells of *Lactiplantibacillus plantarum* AM2 adhered to stainless steel chips visualized by SEM ×5000 (**A**) and ×10,000 (**B**). Percent hydrophobicity of *Lactiplantibacillus plantarum* AM2 to different hydrocarbons (**C**).

The adhesion of *Lactiplantibacillus plantarum* AM2 to stainless steel chips is illustrated in Figs. 3A and 3B. The strain adhered to stainless steel chips by forming clumps without the involvement of specific receptors or fibril as revealed by the images of SEM (Figs. 3A and 3B).

#### Cell surface hydrophobicity of Lactiplantibacillus plantarum AM2

The results presented in Fig. 3C revealed that *Lactiplantibacillus plantarum* AM2 has a maximum hydrophobicity (34.3%) toward hexadecane while the minimum hydrophobicity (19.5%) was observed against octane. The strain also showed a relatively moderate hydrophobicity (28.8%) toward xylene.

#### Antimicrobial activity of *Lactiplantibacillus plantarum* AM2

As shown in Table 5, the culture supernatant obtained from *Lactiplantibacillus plantarum* AM2 was inhibitory to both Gram-positive and Gram-negative bacterial pathogens, however, the strain showed no detectable inhibition against *Candida albicans* (ATCC 10231). The greatest antimicrobial activity of *Lactiplantibacillus plantarum* AM2 (2.86 AU) was against *Escherichia coli* (ATCC 25922). *Salmonella typhimurium* (ATCC 14028), *Pseudomonas aeruginosa* (ATCC 27853), and *Enterococcus faecalis* (ATCC 29212) were effectively inhibited by AM2 strain, while the least antimicrobial activity (1.77 AU) was against *Staphylococcus aureus* (ATCC 29223). On the other hand, neutralization of the *Lactiplantibacillus plantarum* AM2 culture supernatant eliminated its antimicrobial activity against the tested indicator pathogens.

**Table 5:** The antimicrobial activity of *Lactiplantibacillus plantarum* AM2 against human pathogens.

Indicator pathogenic strain	Antimicrobial activity of *Lactiplantibacillus plantarum* AM2 (AU)
*Salmonella typhimurium* (ATCC 14028)	2.77
*Pseudomonas aeruginosa* (ATCC 27853)	2.77
*Enterococcus faecalis* (ATCC 29212)	2.77
*Staphylococcus aureus* (ATCC 29223)	1.77
*Escherichia coli* (ATCC 25922)	2.86
*Candida albicans* (ATCC 10231)	0.0

#### Antibiotic susceptibility of *Lactiplantibacillus plantarum* AM2

Table 6 shows that *Lactiplantibacillus plantarum* AM2 is sensitive to amoxicillin, amoxicillin/clavulanic acid, piperacillin, piperacillin/tazobactam, ceftazidime, cefepime, aztreonam, imipenem, meropenem, amikacin, gentamicin, tobramycin, ciprofloxacin, minocycline, and trimethoprim/sulfamethoxazole.

**Table 6:** Antibiotic suseptibility profile of *Lactiplantibacillus plantarum* AM2.

Antibiotic	Minimum inhibitory concentration (μg/mL)	Susceptibility of *Lactiplantibacillus plantarum* AM2 to the antibiotic
Amoxicillin	8.0	Sensitive
Amoxicillin/clavulanic acid	8.0	Sensitive
Piperacillin	4.0	Sensitive
Piperacillin/tazobactam	4.0	Sensitive
Ceftazidime	1.0	Sensitive
Cefepime	1.0	Sensitive
Aztreonam	1.0	Sensitive
Imipenem	1.0	Sensitive
Meropenem	0.25	Sensitive
Amikacin	2.0	Sensitive
Gentamicin	1.0	Sensitive
Tobramycin	1.0	Sensitive
Ciprofloxacin	0.25	Sensitive
Minocycline	1.0	Sensitive
Trimethoprim/sulfamethoxazole	20.0	Sensitive

### Effect of *Lactiplantibacillus plantarum* AM2 and D-galactose and their combination on time latency of rats in MWM experiment

In this behavioral experiment the rats in D-galactose (D-gal) group showed substantial increase (P < 0.05) in time latency through three phases of experiment compared to control group, on the other hand, combination (*Lactiplantibacillus plantarum* and D-gal) group showed notably decrease (P < 0.05) in time latency in three phases compared to D-gal group (Table 7).

**Table 7:** Effect of *Lactiplantibacillus plantarum* AM2 and D-galactose and their combination on time latency of rats in MWM experiment.

*Parameters*	*Experimental groups*			
	Control group	*L. plantarum* group	D-gal group	*L. plantarum* and D-gal group
First latency time (sec.)	65.40 ± 0.94	63.50 ± 0.90^b^	86.30 ± 1.33^a^	75.40 ± 0.53^ab^
Second latency time (sec.)	57.70 ± 1.13	56.80 ± 1.05^b^	91.20 ± 0.88^a^	74.50 ± 1.40^ab^
Third latency time (sec.)	42.30 ± 0.87	40.90 ± 1.18^b^	91.10 ± 1.00^a^	66.50 ± 1.38^ab^

Values are expressed as means ± S.E.; n = 6 for each group.

^a^The mean values are significantly different compared to the control group at p < 0.05.

^b^The mean values are significantly different compared to the D-gal-group at p < 0.05.

### Biochemical analysis

#### Effect of *Lactiplantibacillus plantarum* AM2 and D-galactose and their combination on serum glucose and AGEs levels of rats

Table 8 shows a noticeable increase in glucose and AGEs levels in D- galactose treated group (P < 0.05) compared to control group, on the other hand the administration of probiotic AM2 in combination with D-gal lead to substantial decrease (P < 0.05) compared to D-gal treated group. While *Lactiplantibacillus plantarum* treated group revealed significant improvement in glucose and AGEs levels respectively compared to control and D-gal groups.

**Table 8:** Effect of *Lactiplantibacillus plantarum* AM2 and D-galactose and their combination on serum glucose and AGEs levels, oxidative stress markers and antioxidant capacity, ACh level and AChE activity in hippocampus, inflammatory markers, amyloid β (Aβ1–42) and caspase-3 in hippocampus of rats.

Parameters	Experimental groups			
	**Control group**	***L. plantarum* group**	**D-gal group**	***L. plantarum* and D-gal group**
Glucose (mg/dL)	77.93 ± 0.35	68.73 ± 2.49^ab^	143.24 ± 3.90^a^	93.82 ± 2.14^ab^
AGE (ng/mL)	49.38 ± 1.07	50.62 ± 0.46^b^	81.98 ± 1.58^a^	62.68 ± 0.78^ab^
MDA (nmol/mg.protein)	11.22 ± 0.42	10.28 ± 0.30^b^	26.57 ± 0.83^a^	15.63 ± 0.23^ab^
GSH (nmol/mg.protein)	19.73 ± 0.53	19.87 ± 0.41^b^	8.08 ± 0.22^a^	15.82 ± 0.28^ab^
Total antioxidant capacity (mM/mg.protein)	1.29 ± 0.01	1.27 ± 0.01^b^	0.74 ± 0.04^a^	1.13 ± 0.02^ab^
ACh (pg/mg.protein)	8.67 ± 0.14	8.92 ± 0.09^b^	3.93 ± 0.14^a^	6.90 ± 0.22^ab^
AChE (pg/mg.protein)	7.77 ± 0.16	8.22 ± 0.07^b^	16.42 ± 0.26^a^	12.27 ± 0.33^ab^
NF-κB (pg/mg.protein)	5.05 ± 0.21	5.22 ± 0.22^b^	18.42 ± 0.29^a^	9.77 ± 0.14^ab^
TNF-α (pg/mg.protein)	117.93 ± 0.88	107.53 ± 1.11^ab^	141.93 ± 2.29^a^	130.33 ± 0.60^ab^
IL-6 (pg/mg.protein)	4.02 ± 0.14	4.17 ± 0.09^b^	9.42 ± 0.32^a^	5.82 ± 0.28^ab^
IL-1β (pg/mg.protein)	31.78 ± 0.50	30.42 ± 0.52^b^	58.28 ± 0.96^a^	40.22 ± 0.60^ab^
Aβ1–42 (pg/mg.protein)	42.42 ± 0.33	40.60 ± 0.48^ab^	70.48 ± 0.90^a^	50.28 ± 0.56^ab^
CASP3 (pg/mg.protein)	57.40 ± 0.51	54.38 ± 0.74^ab^	92.98 ± 1.35^a^	65.05 ± 0.93^ab^

Values are expressed as means ± S.E.; n = 6 for each group.

^a^The mean values are significantly different compared to the control group at p < 0.05.

^b^The mean values are significantly different compared to the D. gal-group at p < 0.05.

#### The effect of *Lactiplantibacillus plantarum* AM2 and D-galactose and their combination on oxidative stress markers and total antioxidant capacity of rats

As shown in Table 8, D-gal group indicated notably increase in MDA levels and significant decrease (P < 0.05) in GSH and total antioxidant capacity compared to control group. While *Lactiplantibacillus plantarum* and D-gal treated group revealed substantial decrease (P < 0.05) in MDA levels and noticeable augment (P < 0.05) in GSH and total antioxidant capacity compared to D-gal treated group.

#### Effect of *Lactiplantibacillus plantarum* AM2 and D-galactose and their combination on ACh level and AChE activity in hippocampus

The results of ACh and AChE revealed considerable decline in ACh level and elevation in AChE activity (P < 0.05), respectively in D-gal treated group compared to control group. Conversely, *Lactiplantibacillus plantarum* and D-gal treated group indicated marked rise (P < 0.05) in ACh level and diminution in AChE compared to D-gal group as shown in Table 8.

#### Effect of *Lactiplantibacillus plantarum* AM2, D-galactose and their combination on inflammatory markers, Amyloid β (Aβ1–42) and CASP-3 in hippocampus of rats

The levels of NF-κB, TNF-α, IL-6, IL-1β, Aβ1–42 and CASP-3 are markedly (P < 0.05) elevated in D-gal treated group compared to control group. Meanwhile, these markers are considerably improved in *Lactiplantibacillus plantarum* and D-gal group and *Lactiplantibacillus plantarum* group (P < 0.05) compared to D-gal and control groups, respectively as shown in Table 8.

### Effect of *Lactiplantibacillus plantarum* AM2, D-galactose and their combination on the histopathological structure of the hippocampus (dentate gyrus) of rats

**Fig. 4:**
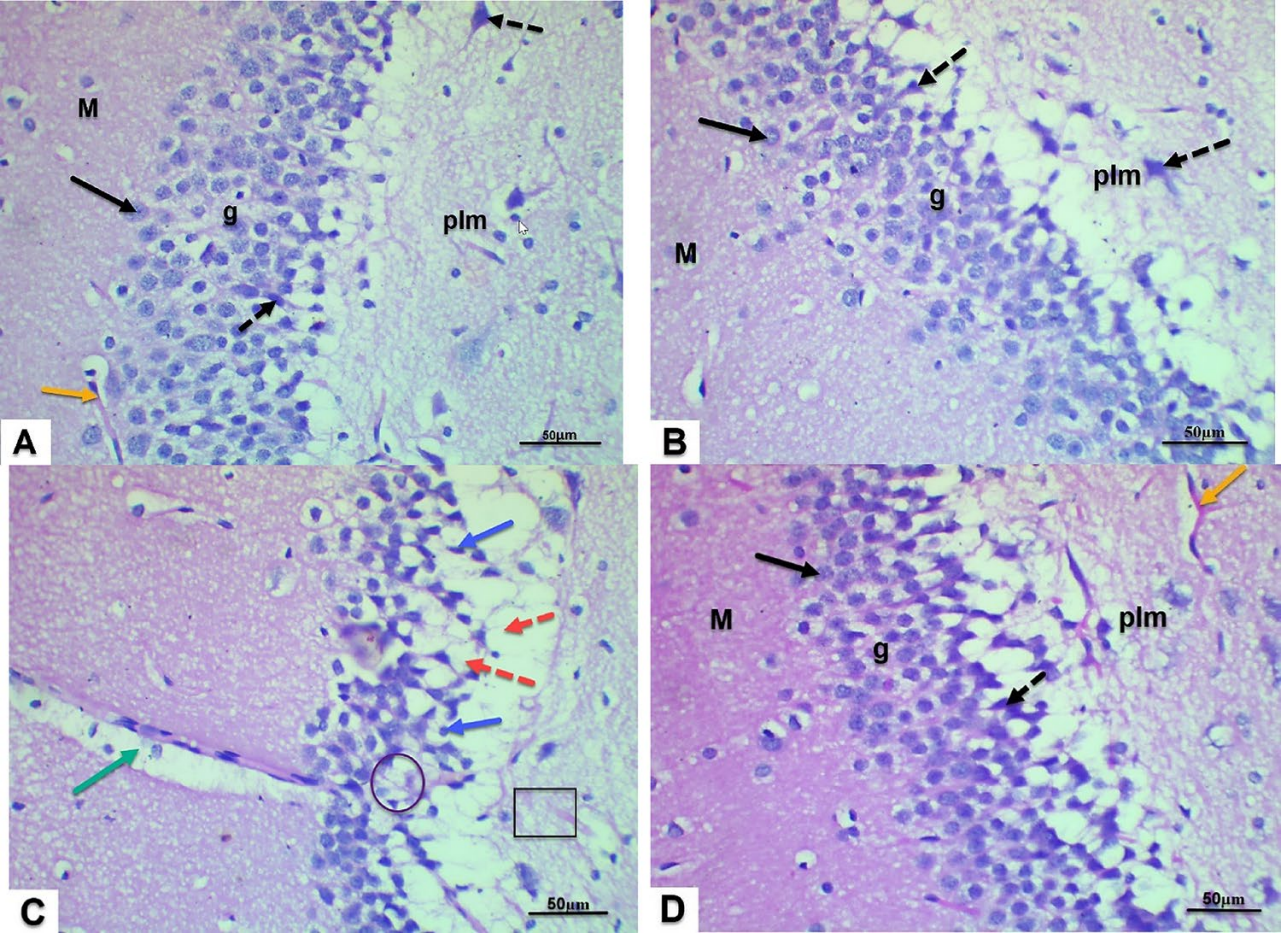
Photomicrographs of the dentate gyrus region within the rat hippocampus. (H & E, ×﻿400). Panels **A** & **B** (control and *Lactiplantibacillus plantarum*-treated animals) display normal architecture with distinct molecular (M) and granular (g) layers. The polymorphic (Plm) layer appears normal, and the granular layer contains numerous healthy granular (black arrow) and pyramidal cells (black dotted arrow). Panel **C** (D-gal) shows a D-gal-treated section with a sparse granular layer, pyknotic nuclei (blue arrow), perinuclear halos (red arrow), and a dilated blood vessel (green arrow), indicating neuronal degeneration (black circle) and encephalomalacia (black square). Panel **D** (*Lactiplantibacillus plantarum* and D-gal) reveals a noticeable improvement with increased intact granular (black arrow) and pyramidal cells (black dotted), and a normal blood vessel (orange). The M and g layers appear healthy.

Figure 4 (A-D) presents histological changes of the rat dentate gyrus from different experimental treatments revealing distinct treatment effects on hippocampal morphology. The dentate gyrus of control and *Lactiplantibacillus plantarum*-treated group displayed a well-defined trilaminar structure, including the molecular layer, granular layer, and polymorphic layer. Additionally, normal vascularity was observed. Contrariwise, D-gal intoxication induced significant alterations within the dentate gyrus. The granular layer exhibited a decrease in cellularity with pyknotic nuclei, characterized by hyperchromatic staining. Perinuclear halos, indicative of cellular injury, were evident alongside dilated blood vessels within the molecular layer. On the other hand, co-treatment with *Lactiplantibacillus plantarum* and D-gal demonstrated a partial amelioration of D-gal-induced damage. The dentate gyrus showed a modest improvement in architecture, evidenced by an increased presence of morphologically normal granular cells. However, some cells still exhibited pyknotic nuclei.

## Discussion

Alzheimer’s disease (AD) is a combination of environmental and genetic factors that trigger its onset. Recently, dysbiosis of gut flora has been linked to the critical role of inflammation and consequently the progression and impact of AD^47^. The gut microbiota communicate with the central nervous system (CNS) through the nervous system or through chemical substances that can cross the blood brain barrier (BBB)^47^. The vagus nerve specifically connects neurons in the CNS with those in the intestine. The gastrointestinal microbiota produces neurochemicals such as GABA, noradrenaline, serotonin, dopamine and acetylcholine (ACh) that can affect the activity of central neurons impacting behaviour^48^. On the other hand, the gut microbiota responds to the brain signals via neurotransmitters^49^. Therefore, the microbiome-gut brain axis (MGBA) is a multifunctional network, where gut microbiota, CNS, endocrine system, immune system participate to the bidirectional communication^6^. One of the most prevalent genus of gut microbiota is *Lactiplantibacillus*^47^. Accordingly, the current study was undertaken with the major aim of selecting and identifying a competent *Lactiplantibacillus* sp. capable of producing ACh to ameliorate neurodegeneration in rats.

As an orientation step, nine different bacterial isolates were isolated from mixed vegetable pickle, sauerkraut, yogurt, and rayeb then screened for their ability to produce ACh. Screening results revealed that only six *Lactiplantibacillus* spp. showed competence to produce ACh. *Lactiplantibacillus* sp. AM2 was selected as the most potent *Lactiplantibacillus* isolate as it showed the highest production of ACh. Isolation of *Lactiplantibacillus* spp. from mixed vegetable pickle, sauerkraut, yogurt, and rayeb was reported in other studies^50–52^.

The most promising *Lactiplantibacillus* isolate AM2 was chosen for further identification using 16S rRNA sequence analysis. Sequencing of PCR-product and comparison of 16S rRNA sequences has been previously reported among type strains of *Lactiplantibacillus*^53–55^. The phylogenetic relationships among the new experimental isolate (AM2) and the closely related *Lactiplantibacillus* spp. have been described in the current study and revealed that, strain AM2 was identified as *Lactiplantibacillus plantarum* representing 100% identity. The data obtained by 16S rRNA consist with the morphological and the biochemical characteristics of *Lactiplantibacillus plantarum*. The morphological and the biochemical properties for identification of *Lactiplantibacillus plantarum* up to the genus level have been reported in other studies^53,56^. Based on the results of 16S rRNA sequencing, a high degree of similarity ranged from 99.33% to 100% was observed between *Lactiplantibacillus plantarum* AM2 and its phylogenetically closest species. Thus, matrix-assisted laser desorption ionization time of flight mass spectrometry (MALDI-TOF MS) was used to compare the protein profiles of AM2 cells to reference spectra. MALDI-TOF MS database revealed that, *Lactiplantibacillus plantarum* was the best match species to AM2 strain. These findings are inline with previous research that used MALDI-TOF MS for identification of *Lactiplantibacillus plantarum* isolated from the gut of sea bream^57^. Within the taxonomic framework of the genus *Lactiplantibacillus*, *Lactiplantibacillus plantarum* is part of the facultatively heterofermentative group of Lactobacilli. This species exhibits a diverse and adaptable nature, found in various ecological niches such as dairy, meat, fish, and a multitude of plant fermentations. Numerous strains of *Lactiplantibacillus plantarum* have already served as starter cultures in creating a broad range of fermented foods and beverages known for their appealing taste and texture^58^.

In order to be used as a putative probiotic strain, the level of tolerance of *Lactiplantibacillus plantarum* AM2 to gastric juice components was evaluated *in-vitro*. Prior to reaching the intestines, probiotic bacteria need to survive the journey through the stomach, where the secretion of gastric acid acts as a primary defense mechanism against most ingested microorganisms. At pH 2, the AM2 strain was less viable compared to its resistance at pH 3 which indicates that the presence of low pH values had a strong inhibitory effect. Similarly, the survival of 7 *Lactiplantibacillus plantarum* strains at pH 3 has been reported previously^52^. Some prominent *Lactiplantibacillus* strains have been observed to remain viable when exposed to pH levels ranging from 2.5 to 4.0 but exhibited reduced viability at lower pH values^59,60^. It is worth noting that, while the pH of the human stomach is 1 during fasting, it varies from 2 to 4 after a meal. Therefore, the strong resistance of *Lactiplantibacillus plantarum* AM2 may enable survival in the gastric acidic environment and function effectively.

When assessing the potential of employing lactic acid bacteria as probiotics, it is typically essential to assess their capacity to withstand the impact of bile acids. Bile acids are produced in the liver from cholesterol and then released from the gallbladder into the duodenum in conjugated form, maintaining a concentration in the intestine ranging from 0.3 to 0.5%. Conjugated and deconjugated bile acids are recognized for their *in-vitro* antibacterial activity against both Gram-negative and Gram-positive bacteria such as *Escherichia coli* strains, *Klebsiella* sp., and *Enterococcus* sp.^61^. Bile acids are extensively chemically modified in the colon primarily through microbial activity, undergoing processes such as deconjugation, dehydroxylation, dehydrogenation, and deglucuronidation. *Lactiplantibacillus plantarum* AM2 was found to be resistant to ox-bile at a concentration of 0.3, 0.5, or 1.0% in MRS broth, suggesting that this strain could survive in the small intestine. Moreover, the AM2 strain was found to be more resistant than some other probiotic *Lactiplantibacillus* strains^52,62^ in terms of their high bile salt tolerance level. The cells of *Lactiplantibacillus plantarum* AM2 likely exhibit resistance to bile due to the expression of related proteins^62^.

Under conditions that simulate gastric and pancreatic juice of the human gastrointestinal tract, *Lactiplantibacillus plantarum* AM2 was able to maintain 81.25% and 99.1% of its viability after incubation for 3 h; 80.43% and 97.2% of its viability after incubation for 4 h, respectively. This finding indicates a high probability of passing through the stomach and working efficiently. The survival of 6 *Lactiplantibacillus plantarum* strains in a simulated pancreatic juice (pH 8.0) after incubation for 240 min except for *Lactiplantibacillus plantarum* S56 strain has been reported previously^52^.

The adherence of *Lactiplantibacillus plantarum* AM2 to mucosal surfaces and epithelial cells is an important property for a probiotic strain. Attachment of bacteria to gastric mucosa is a complex multifactorial process that involves epithelial mucus, bacterial surface proteins, and bacterial capsules^63^. However, the adhesion of AM2 to stainless steel chips showed no involvement of receptors or fibril in the attachment of bacterial cells to the stainless steel surface. Instead, the cells of AM2 strain adhered and deposited as aggregates that formed clumps. Furthermore, *Lactiplantibacillus plantarum* AM2 showed hydrophobicity toward hexadecane, octane, and xylene. These findings have been shown *Lactiplantibacillus plantarum* AM2 to be adhesive owing hydrophobic properties related with the adhesion to epithelia^64^. These outcomes are in line with other reports on the hydrophobicity of *Lactiplantibacillus* spp.^31,32^.

*Lactiplantibacillus plantarum* AM2 was sensitive to cell wall synthesis inhibitors including amoxicillin, amoxicillin/clavulanic acid, piperacillin, piperacillin/tazobactam, ceftazidime, cefepime, aztreonam, imipenem, and meropenem. In addition, it was susceptible to DNA replication inhibitors including ciprofloxacin and to protein synthesis inhibitors including amikacin, gentamicin, tobramycin, and minocycline. Furthermore, the AM2 strain was also sensitive to folic acid metabolism inhibitors including trimethoprim/sulfamethoxazole. Similar pattern of antibiotic sensitivity was observed for several *Lactiplantibacllus* spp.^65^. It is important that the potential probiotic strain do not transfer antibiotic resistance genes to gut microbiota. However, some strains of Lactobacilli were reported to possess non-transmissible, chromosomally encoded, intrinsic resistance to vancomycin^66^, penicillin and streptomycin^67^, polymyxin B, gentamicin, and kanamycin^68^.

The antimicrobial activity of *Lactiplantibacillus plantarum* AM2 against indicator pathogenic strains varied in activity against *Escherichia coli* (ATCC 25922), *Salmonella typhimurium* (ATCC 14028), *Pseudomonas aeruginosa* (ATCC 27853), *Enterococcus faecalis* (ATCC 29212), and *Staphylococcus aureus* (ATCC 29223). However, no detectable antifungal activity was observed against *Candida albicans* (ATCC 10231). In other studies, 17 *Lactiplantibacillus plantarum* strains showed different antibacterial activity against *Escherichia coli* and *Staphylococcus aureus*^69^; several *Lactiplantibacillus plantarum* strains were active against *Salmonella typhimurium* and other selected indicator pathogens^70–72^. As observed in the current study, the inhibitory activity of the *Lactiplantibacillus plantarum* AM2 strain was eliminated after neutralization with alkali. Accordingly, the antagonistic activity of the AM2 strain might be due to synthesis of organic acids in the culture supernatant. Similarly, the inhibitory effect of some *Lactiplantibacillus* strains against Gram negative pathogens was attributed to their synthesis of organic acids^73^. Lactobacilli have been also reported to produce fatty acids, hydrogen peroxide, inhibitory metabolites, bacteriocins, and analogous bacteriocins with different antibacterial activity^60,74^.

In the current study, *Lactiplantibacillus plantarum* AM2 was evaluated for its potential to ameliorate neurodegeneration in Wistar albino rats. The group of rats treated with D-galactose (D-gal) for AD induction showed an increase in time latency across three phases of Moris water maze compared to the control group, which may be due to induction of aging processes by D-gal administration^75^. However, the administration of the probiotic *Lactiplantibacillus plantarum* AM2 alongside D-gal resulted in a decrease in time latency during these phases compared to the D-gal group, indicating the probiotic’s potential to mitigate cognitive dysfunction^76^.

Our results also revealed that D-gal administration significantly elevated levels of advanced glycation end-products (AGEs) and glucose compared to the control group. These findings are consistent with those of another study^77^, which reported that D-gal increases AGEs as oxidative products and that the elevated glucose levels contribute to neuro-inflammation, which in turn promotes neurodegeneration. Conversely, the group treated with *Lactiplantibacillus plantarum* showed a notable decrease in AGEs and glucose levels compared to the D-gal group, likely due to the antioxidant properties of the AM2 strain, which helps reduce oxidative stress and neurodegeneration. The antioxidant capacity of *Lactiplantibacillus plantarum* was previously highlighted in other study^78^, which reported that *Lactiplantibacillus plantarum* NCIMB 8826 produces significant amounts of antioxidants, such as ferulic acid.

Various biomarkers were analyzed from hippocampal homogenates in this study to assess their levels in both disease and healthy states. These biomarkers included MDA, GSH, TAC, ACh, AChE, NF-κB, TNF-α, IL-6, IL-1β, Aβ, and CASP-3. Our findings indicated disturbances in D-gal-treated rats, characterized by increased hippocampal MDA levels and decreased GSH and TAC, suggesting an overproduction of free radicals in the tissues, leading to oxidative stress and an imbalance between the antioxidant system and ROS^79^. Similarly, another study^80^ reported that D-gal causes an increase in MDA levels, a marker of oxidative stress, and a decrease in antioxidant levels such as GSH and TAC. On the other hand, treatment with *Lactiplantibacillus plantarum* AM2 significantly reduced MDA levels while improving GSH and TAC levels in the hippocampus. This effect is attributed to the antioxidant properties of the AM2 strain, which helps restore balance between antioxidants and ROS^81^.

ACh is a key neurotransmitter with a crucial role in cognitive functions, particularly memory and learning. AChE plays an essential role in the cholinergic system by terminating neuronal signal transmission at synapses, preventing the diffusion of ACh and the activation of nearby receptors. In the current study, the D-gal group showed a significant decrease in ACh levels and a marked increase in AChE levels compared to the control group, which are indicators of neurodegenerative disorders due to the loss of synapses in cholinergic neurons^82^. Another study also reported that D-gal negatively affects ACh levels, leading to an increase in AChE activity^83^. However, the combination of *Lactiplantibacillus plantarum* and D-gal noticeably improved both ACh and AChE levels, possibly due to *Lactiplantibacillus plantarum*’s protective effects on hippocampal tissue against cholinergic dysfunction, aiding recovery from AD^36^.

Pro-inflammatory cytokines and the factors regulating their production play a vital role in neurodegenerative diseases^5^, contributing to the accumulation of Aβ and the activation of CASP-3. In the D-gal group, the significant increases in hippocampal NF-κB, TNF-α, IL-6, IL-1β, Aβ, and CASP-3 could be attributed to excessive ROS production which leads to energy imbalance and oxidative stress-mediated NF-κB activation, promoting pro-inflammatory cytokine production and subsequent behavioral decline^84^. Additionally, pro-inflammatory cytokines contribute to neuro-inflammation, which induces Aβ accumulation and increases CASP-3 levels^85^. Elevated ROS levels in D-gal-treated rats may also activate CASP-3, a key mediator of apoptosis, leading to neuronal degeneration and consequently, oxidative stress and inflammation drive neuronal apoptosis^86^. In contrast, supplementation with the AM2 strain in combination with D-gal effectively regulated the production of inflammatory biomarkers and CASP-3 compared to the D-gal group. Previous studies have shown that *Lactiplantibacillus* exhibits anti-inflammatory effects by reducing the production of NF-κB, TNF-α, IL-6, IL-1β, Aβ, and CASP-3^87,88^.

To further confirm these findings, the histopathological features of hippocampal tissue from all experimental groups were examined. As expected, the D-gal-treated group exhibited various alterations, including reduced cellularity in the granular and polymorph layers with pyknotic nuclei, hyperchromatic staining, perinuclear halos indicative of cellular injury, and dilated blood vessels in the molecular layer. These observations align with previous studies that have reported the harmful effects of D-gal on the brain^89,90^. The observed hippocampal tissue damage is consistent with the notion that D-gal-induced oxidative stress contributes to lipid peroxidation and subsequent neuronal degeneration^91^. In contrast, treatment of D-gal rats with AM2 demonstrated a balance between oxidants and antioxidants, highlighting the modulatory roles of probiotics on inflammatory markers^92^. As revealed in the current study, the AM2 strain ability to delay neurodegeneration likely occurs through various mechanisms including the regulation of choline metabolism and production of ACh which may enhance cholinergic neurotransmission, modulation of inflammation, regulation of apoptosis, inhibition of D-gal-induced overexpression of AChE, and the inhibition of Aβ deposition.

## Conclusion

*Lactiplantibacillus plantarum* AM2 demonstrated promising probiotic properties and the ability to produce acetylcholine. Additionally, the strain effectively mitigated neurodegeneration in an *in-vivo* study using Wistar albino rats with Alzheimer’s disease induced by D-galactose. Future studies should prioritize clinical trials to validate its effectiveness in managing Alzheimer’s disease in humans and investigate the mechanisms underlying acetylcholine-mediated neuroprotection.

## Data Availability

The datasets supporting the conclusions of this article are included within the article.
